# Fertility rates among very young adolescent women: temporal and spatial trends in Brazil

**DOI:** 10.1186/s12884-016-0843-x

**Published:** 2016-03-18

**Authors:** Ana Luiza Vilela Borges, Christiane Borges do Nascimento Chofakian, Ana Paula Sayuri Sato, Elizabeth Fujimori, Luciane Simões Duarte, Murilo Novaes Gomes

**Affiliations:** School of Nursing, University of São Paulo, Av. Dr. Enéas Carvalho de Aguiar 419, Cerqueira César, CEP 05403-000 São Paulo, SP Brazil; Department of Epidemiology, Faculty of Public Health, University of São Paulo, São Paulo, Brazil; CDA-Agricultural Defense Coordination, São Paulo, Brazil

**Keywords:** Adolescent, Very young adolescent, Birth rate, Age-specific fertility rate, Adolescent pregnancy, Sexual and reproductive health

## Abstract

**Background:**

We assessed whether the reported decrease in fertility rates among 15 to 19 years old Brazilian adolescents has met with a parallel decrease in very young adolescent (10 to 14 years old) fertility rates. So we explored temporal trends for fertility rates among very young adolescents between 2000 and 2012 for Brazil as a whole, its regions and states; and also analyzed the spatial distribution of fertility rates among Brazilian municipalities in the years 2000 and 2012.

**Methods:**

We used data from the Information System on Live Births to calculate the rates. To examine the temporal trends, we used linear regression for time series with Prais-Winsten estimation, including the annual percentage change, for the country, regions, and states. To analyze the spatial distribution among Brazilian municipalities, we calculated the Global Moran Index and created a local Moran significance and cluster map through Local Indicators of Spatial Association (LISA). We also elaborated a thematic map with the rates using empirical Bayesian estimation.

**Results:**

Brazilian very young adolescent fertility rates remained high and stable throughout the 2000 to 2012 period, and significantly decreased in three out of 26 states, and in the federal district. On the other hand, an increase was observed in two Northern and Northeastern states. The rates were spatially dependent in Brazilian municipalities (Moran Index = 0.22 in 2012; *p* = 0.05). The maps indicated a heterogeneous distribution of the rates, with high-rate clusters predominant in the North and low-rate clusters predominant in the South, Southeast, and Midwest.

**Conclusions:**

Our findings indicate that Brazilian very young adolescent fertility rates have not decreased in parallel with adolescent fertility rates as they remain high and did not decrease from 2000 and 2012, even though a few states presented a decrease. Thus, these phenomena probably have distinct underlying causes that warrant further elucidation. Progress in this field is crucial for the development of specific policies and programs focused on very young adolescents.

## Background

Adolescent pregnancy has strong negative effects on the educational and professional opportunities for women [1]. Thus, considerable research and specific actions have targeted this problem. Consequently, the adolescent fertility rate, defined as births per 1,000 among women aged between 15 and 19, has recently declined in the majority of high and middle-income countries, including the USA [2, 3]. Brazil has followed the same trend, with the adolescent fertility rate decreasing from 80.1 in 2000 to 60.9 in 2010 [4]. This decline has occurred with marked regional differences [5], mainly because of income inequality among municipalities [4], much like previously observed for abortion and maternal mortality rates [6, 7].

Where measured, childbirths among very young adolescents (VYA) aged 10 to 14 years occur at much lower rates. For example, while the adolescent fertility rate is 10 in Spain and 34 in the USA, the VYA fertility rates in these countries are only 0.14 and 0.45, respectively [3]. Nevertheless, when a VYA gives birth, consequences are truly damaging. Morbidity and mortality rates increase with decreasing age in this group. In VYA, consequences such as puerperal endometritis, episiotomy, postpartum hemorrhage, operative vaginal delivery, and anemia are more frequent. Moreover, maternal death is 4 times higher than among women aged 20 to 24 years [8].

In face of these drastic consequences and the lack of data, the World Health Organization [9] has emphasized the need for the development of policies, programs, and research targeting sexual and reproductive issues of VYA. In this context, we assessed whether the reported decrease in fertility rates among Brazilian adolescents has met with a parallel decrease in Brazilian VYA fertility rates. To this end, we investigated the temporal trends in VYA fertility rates between 2000 and 2012 for Brazil as a whole, as well as for its regions and states. We also analyzed the spatial distribution of fertility rates among all Brazilian municipalities in the years 2000 and 2012. Our results indicate that, unlike what has been observed among adolescents, VYA fertility rates have not decreased and probably require specific and distinct action both in Brazil and worldwide.

## Methods

**Fig. 1:**
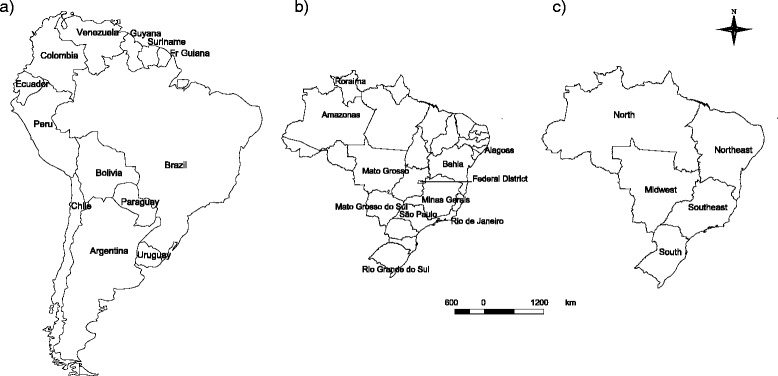
Brazilian geographical features: **a** Brazil and South America; **b** Brazilian states and federal district; **c** Brazilian regions

Brazil is a federative republic that covers almost half of South America (Fig. 1a), with a population close to 200 million [10]. The political and administrative organization of Brazil consists of a federal district (seat of the federal government, with judiciary, legislative, and executive powers), 26 states (the highest ranking unit within the political and administrative organization of the country) (Fig. 1b), and 5,565 municipalities in 2012 (lower unit hierarchy within the political and administrative organization). It is important to point out that the number of municipalities varied across the period of study: it was 5,561 in 2000, but from 2010 it turned out to be 5,565. In addition, Brazil is divided into five geographical regions: North, Northeast, Midwest, Southeast, and South (Fig. 1c) [10].

The country has presented one of the highest levels of income inequality in the world [11]. Even though improvements in social and human development are in progress [12], regional and social inequalities remain, resulting in large internal heterogeneity. This contrast is easily verified when the Human Development Index (HDI) is compared among municipalities. The HDI is a summary measure of achievements in three key dimensions of human development: health, education, and income. It sets a minimum and a maximum range, expressed as a value between 0 and 1. The closer a HDI is to 1, the more achievements in human development the setting has attained. In Brazil, municipalities with higher HDI are located mainly in the South and Southeast. The highest ranked has an HDI of 0.862 and is a Southeastern municipality. In 2010, the North and Northeast regions held a concentration of municipalities with very low or low HDI, including the lowest ranked municipality, with an HDI of 0.418 [13]. Such a context of inequality has been recognized to affect many health outcomes, including those related to adolescent health [14].

### Data source and definitions

We analyzed the live births registered in Brazil from 2000 to 2012. In this period, there were 359,963 live births to women aged 10 to 14 years old, representing 0.93% of all 38,815,012 births at the national level [15].

To estimate the VYA fertility rate, we divided the births to women aged 10 to 14 years by the number of female residents aged 10 to 14 years old. The values are presented in terms of live births per 1,000 adolescents. The numerators of the age-specific fertility rate for the VYA were provided by the Brazilian Information System on Live Births (SINASC), and the denominators were obtained from the National Bureau of Statistics (IBGE). For 2000 and 2010, we used censuses’ data. For the other years, we used projections. Regarding 2011 and 2012, the Ministry of Health/DATASUS applied the age/sex distribution observed in 2010 census to adjust data, since IBGE projected only total population by municipalities [15, 16]. Data is publicly available at the Brazilian Ministry of Health National Health Information System (DATASUS) site (http://www2.datasus.gov.br/DATASUS/index.php).

The study protocol conforms to the ethical guidelines of the 1975 Declaration of Helsinki and was approved by the University of São Paulo School of Nursing, Brazil (no. 170.604).

### Statistical methods

In order to chart the temporal trend between 2000 and 2012, we calculated the VYA fertility rates by running a linear regression for each of the 26 states, the federal district, the five regions (North, Northeast, Midwest, Southeast, and South) and the whole country. Trend analysis was performed in Stata 13.0 using linear regression for time series with Prais-Winsten auto-regression procedure to minimize first-order temporal autocorrelation of residues. The dependent variable was 10-to-14 fertility rates (per 1,000) and the independent variable was the calendar year (2000 to 2012). When the regression coefficient was not significantly different from zero (p > 0.05), the trend was considered stable. When the coefficient was positive or negative, the trend was considered as increasing and decreasing, respectively. We also calculated the annual percent change (APC) and its respective 95% confidence interval (CI).

To analyze the spatial distribution of the fertility rates in the total of municipalities in the years 2000 and 2012, we calculated the rates for each of the 5,565 municipalities of Brazil. In 2000, there were six births with missing information about the birthplace, whereas in 2010 there was only one. These cases were excluded. We used digital maps from 1997 and 2013 available at the National Bureau of Statistics/IBGE [17]. The 1997 digital map was used for the 2000 data. The 2013 digital map was used for the 2012 data and some adjustments were necessary as five municipalities were created in 2013 and did not compile 2012 data. In order to correct this mismatch, the geographical limits of those five municipalities were edited according to their original features. Only one municipality was not available on the 2013 digital map, so we considered its fertility rate to be the same as the municipality from which it was formed.

We conducted spatial analysis in three ways. First, we used Global Moran Index to verify the presence of global spatial dependence on the age-specific fertility rates [18]. Second, we used the Local Indicators of Spatial Association (LISA) for local spatial association analysis, which produces a specific value for each municipality, thus allowing the identification of municipalities groups with similar rates (clusters). We then produced a map representation identifying municipalities whose neighbors have similar values of VYA fertility rate; thus the map is built by categories, with two possible positive spatial association classes (high-high, low-low) and two negative spatial association (high-low, low-high). We consider only the mapping of municipalities with statistically significant autocorrelation (*p* < 0.05). It is important to point out that both Global Moran Index and LISA analysis were conducted with ‘pure’ VYA fertility rates in order not to influence the estimates, even considering instability due to the great variability on the number of local live births and female 10–14 years-old population.

Third, we elaborated maps with local VYA fertility rates estimated with the Empirical Bayesian method, with the purpose of minimizing the bias of local level age-specific rate estimation as large variations are expected among very small municipalities [19–21]. This means that when calculating the age-specific fertility rate of a location, the neighborhood rate is also considered, which converges towards a local rate average. If a given location is highly populated, the rate presents small variability and remains nearly unchanged when compared to its original rate. On the other hand, if the location is sparsely populated, the estimates present great variation and low weight is assigned to the rate, giving the nearest Bayesian rate an expected value of a randomly chosen area. All spatial analysis were done in Terraview 4.2 with the Spatial Contiguity Weight matrix [22].

## Results

### Fertility rates among very young adolescent women remained stable throughout Brazil from 2000 to 2012

**Fig. 2:**
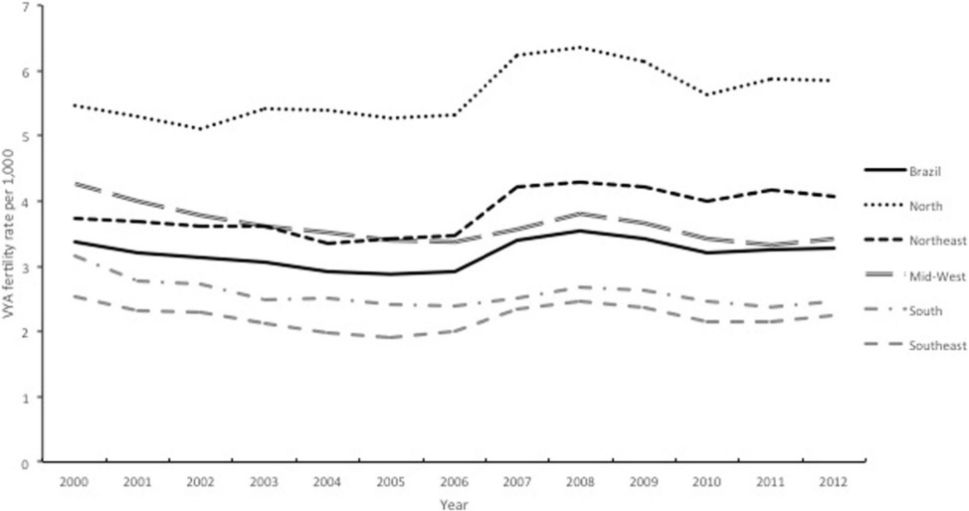
Fertility rates among adolescents between 10 and 14 years of age in Brazil and its geographic regions, 2000-2012

Figure 2 displays fertility rates among women between 10 and 14 years of age in different regions of Brazil as well as in the entire country, from 2000 to 2012. The highest rates were observed in the Northern region and the lowest in the Southeastern region. Trends were similarly stable over time in the individual geographic regions and in the country, with the exception of the Midwest and Northeast that reversed positions for the second highest rate (Fig. 2).

Table 1 shows the fertility rates among VYA women in each Brazilian state in the years 2000 and 2012, as well as Prais-Winsten regression parameters and the average APC for the 2000 to 2012 period. In 2000, the countrywide fertility rate for this group was 3.38, and in 2012 no significant changes to this number were recorded. In fact, rates remained stable in all except in two regions, the Midwest and South, where rates fell significantly. In 2012, at the state level, the highest rate was observed in Roraima (8.77), and the lowest in Minas Gerais (1.94). In 18 out of 26 states, fertility rates were higher than the country average. The states of Amazonas and Bahia had significant positive APC of 2.49% and 1.65%, respectively. On the other hand, only three states including Mato Grosso, Rio de Janeiro, and Rio Grande do Sul, and two regions (Midwest and South), as well as the federal district displayed a significant decrease in fertility rates, varying from −1.59% to −2.82%, annually.

**Table 1:** Fertility rates (years 2000 and 2012) and fertility rate trends (2000 to 2012) among adolescents between 10 and 14 years of age in Brazilian regions and states

	Fertility rate per 1,000		Fertility rate trend 2000 to 2012				
	2000	2012	APC^a^	APC CI 95 %		r^2^	Trend
BRAZIL	3.38	3.29	0.15	−1.48	1.80	0.88	-
Distrito Federal^b^	2.86	2.05	−2.82	−4.38	−1.24	0.83	Decrease
NORTH	5.46	5.85	1.05	−0.15	2.27	0.88	-
Acre	7.42	7.11	0.84	−0.19	1.88	0.58	-
Amapá	6.70	5.63	−0.48	−2.16	1.18	0.04	-
Amazonas^b^	5.57	7.05	2.49	1.33	3.67	0.73	Increase
Pará	4.89	5.56	1.34	−0.36	3.08	0.89	-
Rondônia	4.72	3.50	−1.54	−3.14	0.10	0.40	-
Roraima	10.86	8.77	−1.93	−3.92	0.11	0.06	-
Tocantins	6.20	5.00	1.26	−3.32	0.87	0.88	-
NORTHEAST	3.72	4.07	1.21	−0.42	2.86	0.83	-
Alagoas	4.80	5.63	1.86	−0.85	4.64	0.72	-
Bahia^b^	3.39	3.84	1.65	0.07	3.25	0.74	Increase
Ceará	3.54	3.56	0.57	−1.70	2.90	0.77	-
Maranhão	3.94	4.98	2.42	−0.04	4.94	0.55	-
Paraíba	3.22	3.51	1.01	−0.32	2.35	0.42	-
Pernambuco	3.83	4.07	0.68	−1.21	2.60	0.79	-
Piauí	3.83	3.55	0.18	−1.56	1.94	0.52	-
Rio Grande do Norte	4.30	3.83	−0.60	−3.91	2.83	0.76	-
Sergipe	3.87	3.86	1.03	−2.10	4.25	0.65	-
MIDWEST^b^	4.26	3.41	−1.59	−2.92	−0.24	0.95	Decrease
Goiás	3.69	3.07	−1.25	−3.09	0.63	0.87	-
Mato Grosso^b^	5.27	3.99	−1.70	−2.75	−0.63	0.88	Decrease
Mato Grosso do Sul	5.54	4.86	−0.50	−1.91	0.92	0.78	-
SOUTHEAST	2.54	2.24	−0.64	−2.83	1.60	0.74	-
Espírito Santo	2.96	2.73	−0.35	−3.47	2.86	0.64	-
Minas Gerais	1.99	1.94	0.08	−1.66	1.85	0.64	-
Rio de Janeiro^b^	3.60	2.81	−1.90	−3.37	−0.42	0.89	Decrease
São Paulo	2.41	2.13	−0.38	−3.02	2.34	0.61	-
SOUTH^b^	3.15	2.45	−1.59	−3.04	−0.12	0.88	Decrease
Paraná	3.32	2.93	−0.77	−2.22	0.70	0.85	-
Rio Grande do Sul^b^	3.39	2.14	−2.76	−3.84	−1.68	0.72	Decrease
Santa Catarina	2.41	2.13	−0.71	−2.87	1.51	0.80	-

^a^APC = annual percent change

^b^Significant at level 0.05

### Fertility rates among very young adolescent women was spatially dependent and heterogeneously distributed among Brazilian municipalities in the years 2000 and 2012

**Fig. 3:**
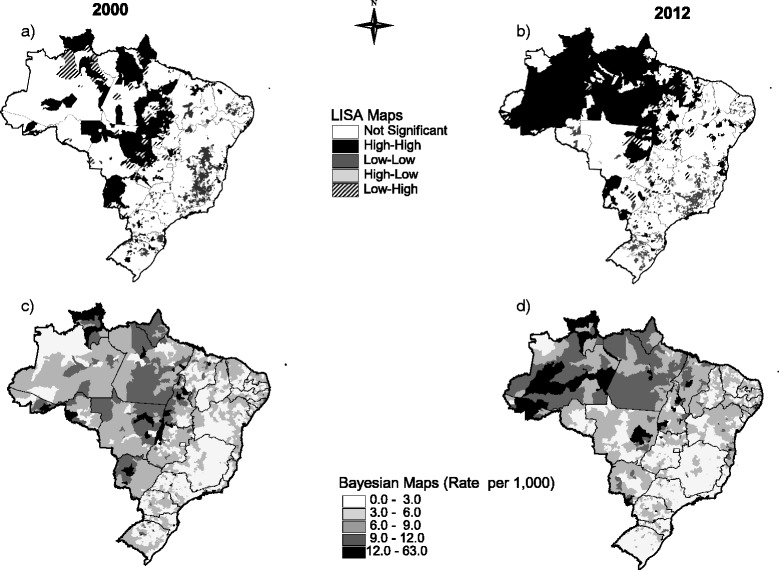
LISA (panels **a** and **b**) and Bayesian thematic maps (panels **c** and **d**) showing the distribution of fertility rates among adolescents between 10 to 14 years old across Brazilian municipalities, in the years 2000 and 2012

We calculated the Global Moran Index and elaborated thematic maps to assess the distribution of fertility rates in our group of interest in all Brazilian municipalities (Fig. 3a-d) in the years 2000 and 2012. The Global Moran Index was 0.19 and 0.22 (*p* = 0.05) for 2000 and 2012, respectively. These results show that fertility rates among the VYA are spatially dependent. In 2000, the LISA map (Fig. 3a) indicates a sparse distribution of high-rate areas (high-high clusters) in the North and Midwest. Low-low clusters were mainly observed in the state of Minas Gerais, Southeastern region, and in the Southern region. In 2012, high-high clusters remain predominant in the Northern region; on the other hand, there is an increase in areas with low rates in the Southern region (Fig. 3b).

The thematic maps where Bayesian estimates were applied yielded similar results in comparison with LISA maps (Fig. 3c-d). Comparing the year 2000 to year 2012, we observed that there was an increase in high-rate areas in the North and Northeast, while a decrease occurred in the South and Midwest regions. In 2012, the highest rates were observed in the North region, including the state of Amazonas, some sparse areas along the borders with Paraguay and Bolivia, as well as in tourism-intensive areas in the Northeastern region, including the southern parts of the states of Bahia and Alagoas.

## Discussion

Pregnancies and childbirths among VYA have drastic health and social consequences. Yet, scant data exist on the subject. Thus, the present study focused on VYA fertility rate trends in Brazil. Our results indicate that, overall, these rates remained stable between 2000 and 2012. The spatial distribution pattern remained heterogeneous depending on the region, state, or municipality.

Brazil has a high VYA fertility rate (3.29 per 1,000) when compared to high-income countries such as Switzerland, Sweden, and New Zealand, where rates vary from 0.01 to 0.17 [3]. Specific factors may determine high countrywide rates in Brazil such as poverty, low educational level, and the relatively poor quality of health services [23]. However, differences may also stem from the high proportion of adolescent pregnancies that end in abortions in high-income countries [3]. In Brazil, abortion is legally restricted, which means that VYA may be more likely to maintain their pregnancies. On the other hand, Brazilian rates lie far below those of some African and Asian countries [24] where early marriages are common, translating into higher VYA fertility rates [25, 26].

Sexual violence or coercion towards VYA may also play a role in determining the high fertility rate of this group in Brazil, as elsewhere. Intercourse in the VYA age group is more likely to be nonconsensual [27], and the relation between sexual abuse and adolescent pregnancy is strongly supported [28]. Because of its developmental stage, the VYA group is also more exposed to the risks and consequences of nonconsensual intercourse [29], including unwanted pregnancies, sexually transmitted diseases, and induced abortion [30, 31]. Therefore, the country needs to make efforts in order to assure VYA rights, so they can have a full and safe development towards adulthood. Prevention and detection of violence and abuse is certainly a strategy to achieve such goal.

Some significant decreases in VYA fertility rates were observed in regions and states with higher HDI including the South and Midwest regions, the states of Mato Grosso, Rio de Janeiro, and Rio Grande do Sul, and the federal district. Because the HDI integrates income, health and education indicators [13], it reflects local and regional investments in improvement of living conditions with direct effects on the future of adolescents. When adolescent women have positive economic prospects, they can invest in human capital, thus preventing or postponing motherhood [32, 33].

The Midwest region displayed a significant decrease in fertility rates. However, one of the states of this region, where the rates remained stable – Mato Grosso do Sul – showed cluster areas formed in border regions with Paraguay and Bolivia. Border areas usually differ from other locations, because nationalities, cultures, and customs mingle. Moreover, border regions favor drug trafficking and the sexual exploitation of children and adolescents. This context may contribute to the occurrence of VYA pregnancies, as it does regarding the spread of HIV/AIDS [34, 35].

Despite these scattered decreases in fertility, the high VYA fertility rates detected in the country and the increases observed in two states (Amazonas and Bahia) represent an important social concern. Amazonas is the largest Brazilian state, and most of its area is covered by the Amazon Forest. More than 178 thousand indigenous people live in protected reservations or unprotected areas [36]. The strong presence of indigenous groups may explain why the state VYA fertility rate (as well as other Northern states) is high even for Brazilian standards. Total fertility rates are three times higher among indigenous than non-indigenous women [37]. However, the increase in VYA fertility rate in Amazonas can be partly attributed to improvements in vital statistics notifications observed in the state and in the entire Northern region in the last decades, even though birth underreporting still occurs [38]. Furthermore, we cannot refute that such improvement in the coverage of the Brazilian System on Live Births Information/SINASC may have influenced these results. If stable or increasing VYA fertility rates are somehow due to a combination of decrease in fertility and an improvement in the coverage, is not known and requires further investigation.

Sexual tourism may explain the increase in VYA fertility rate observed in the state of Bahia. The map shows an increase in high-rate clusters in the south of the state, coinciding with the location of touristic municipalities. A study conducted at touristic seaside municipalities in the states of São Paulo and Rio de Janeiro indicated that tourism has some negative impacts on host communities, which become more susceptible to risky sexual behaviors and elevated alcohol use [39]. The same work shows that the inequality between higher income tourists – some from abroad – and less educated and poorer young women poses a barrier for local adolescent empowerment.

The states of Amazonas and Bahia also underperform in terms of investments in VYA education. The proportion of 15- to 17-year-old adolescents who finished middle school is less than 45%, similarly to other Northern and Northeastern states [40]. Delayed school progression and school abandonment represent problems that often underlie adolescent pregnancy [1] because they undermine women’s empowerment of their own lives. A deep connection with the school network more effectively changes behavior than increasing the knowledge and individual skills around health issues [9, 41, 42]. Thus, access to school is one of the most important factors influencing global adolescent health [14]. Moreover, school attendance is associated with later age of marriage, better use of contraceptives, and greater access to health information [41].

The analysis of the spatial distribution of fertility rates among Brazilian municipalities reiterates the pattern, with a concentration of higher rates in the Northern region, including the state of Amazonas, and some areas in the Northeast. These findings indicate that births among VYA may be associated with income inequality much as observed for older adolescents [4]. Despite important improvements in health status, including improvements in the access to family planning methods [43], and rapid economic, social, and environmental change, Brazil still is a large country with important social and regional inequalities [23].

### Limitations

Some limitations of our study should be pointed out. The rate denominators (number of girls aged 10–14) are intercensal estimates based on trends analysis (except for the years 2000 and 2010) and could influence the results. The use of secondary data from the Brazilian Information System on Live Births/SINASC may have inconsistencies, especially because reliability and underreporting may differ significantly among regions. Anyway, SINASC currently covers more than 90% of births in the whole country and shows substantial improvement in the last decade, especially in Northern and Northeastern regions [15, 38]. Regarding the quality of information around the mother’s age, for instance, studies showed a very low level of missing data in the country, which was 1% in 2005, and almost equal to zero since then [15, 44, 45].

## Conclusions

Our results provide an ecological perspective. Additional research should address Brazilian VYA to generate demographic data on sexual and reproductive health such as age of first intercourse, contraceptive use, pregnancy, delivery and maternal morbidity and mortality, including ethnicity issues. Moreover, further research should be interdisciplinary, also covering cultural values, educational and professional perspectives, family environment, and connectedness to school.

Our findings indicate that Brazilian VYA fertility rates have not decreased in parallel with adolescent fertility rates. Thus, these phenomena probably have distinct underlying causes that warrant further elucidation. Progress in this field is crucial for the development of specific policies and programs focused on VYA.

### Ethics approval and consent to participate

The study protocol conforms to the ethical guidelines of the 1975 Declaration of Helsinki and was approved by the University of São Paulo School of Nursing, Brazil (no. 170.604).

### Availability of data and materials

Data supporting our findings are of open access and can be found at DATASUS (http://www2.datasus.gov.br/DATASUS/index.php) and IBGE (http://www.ibge.gov.br/home/).

## Abbreviations

APC: annual percent change
CI: confidence interval
HDI: human development index
IBGE: National Institute of Statistics (Instituto Brasileiro de Geografia e Estatística)
LISA: Local Indicators of Spatial Association
SINASC: Brazilian Information System on Live Births (Sistema de Informações sobre Nascidos Vivos)
VYA: very young adolescents
