# Erratum to: Fertility rates among very young adolescent women: temporal and spatial trends in Brazil

**DOI:** 10.1186/s12884-016-0977-x

**Published:** 2016-08-03

**Authors:** Ana Luiza Vilela Borges, Christiane Borges do Nascimento Chofakian, Ana Paula Sayuri Sato, Elizabeth Fujimori, Luciane Simões Duarte, Murilo Novaes Gomes

**Affiliations:** 1 School of Nursing, University of São Paulo, Av. Dr. Enéas Carvalho de Aguiar 419, Cerqueira César, CEP 05403-000 São Paulo, SP Brazil; 2 Department of Epidemiology, Faculty of Public Health, University of São Paulo, São Paulo, Brazil; 3 CDA-Agricultural Defense Coordination, São Paulo, Brazil

## Erratum

After publication of the original article [1], it came to the authors’ attention that a source of funding for the study was inadvertently omitted from the Acknowledgements section. The authors would like to add the following statement in the Acknowledgements section:

Publication of this article was funded by the São Paulo Research Foundation (FAPESP) (process 2016/03933-6).

## References

[1] Borges ALV, Chofakian CBN, Sayuri Sato AP, Fujimori E, Duarte LS, Gomes MN (2016). Fertility rates among very young adolescent women: temporal and spatial trends in Brazil. BMC Pregnancy Childbirth.

